# The tensile behaviour of paper under high loading rates

**DOI:** 10.1007/s10570-024-06266-0

**Published:** 2024-12-03

**Authors:** Georg Baumann, Caterina Czibula, Ulrich Hirn, Florian Feist

**Affiliations:** 1 Vehicle Safety Institute, Graz University of Technology, Inffeldgasse 13/6, 8010 Graz, Austria; 2 Institute of Bioproducts and Paper Technology, Graz University of Technology, Inffeldgasse 23, 8010 Graz, Austria

**Keywords:** Paper, Split Hopkinson bar, Strain-rate dependency, Tensile testing

## Abstract

This work deals with the strain-rate dependent characterization of paper under uniaxial tension at high strain-rates. Experiments were performed involving a Split Hopkinson bar for high strain-rate testing, comparing the results with conventional quasi-static tests. Tests were conducted in a strain-rate range between 0.0083 and 212 s^−1^, which is equivalent to testing velocities between 0.0003 and roughly 13.6 m/s. For the first time the change in tensile behaviour of paper is comprehensively characterized and modelled, using the Cowper-Symonds model for strain-rate hardening. The experimental tests showed that the tensile strength as well as the initial stiffness were gradually increasing with increasing strain-rate. The increase in tensile strength between the lowest and the highest strain-rate was 58% on average whereas the mean increase in stiffness between these two strain-rates was almost 115%. Regarding the fracture strain, it was observed that it significantly decreases with increasing strain-rate. While the average fracture strain of the quasi-static tests was at roughly 6% it was close to 3% for the dynamic tests. In case of the Split Hopkinson bar tests, high-speed videos of the samples were made to determine their elongation via target tracking and digital image correlation (DIC). We found that strain localization, which is a highly relevant mechanism for quasi-static tensile failure, is likely related to short term plastic creep of the material as strain localization nearly entirely disappears at high loading rates of paper.

## Introduction

### Motivation

During their lifetime, paper products are subjected to a wide range of load rates, covering several orders of magnitude from creep over quasi-static to impact. In packaging applications, paper and pulp products can be under constant stress for a prolonged time. During processing and manufacturing, however, the same products can be exposed to high load rates. In conventional processing operations, such as roll-to-roll manufacturing, creasing, folding or calendaring pulling speeds of up to 20 m/s (Holik 2006) are commonly applied and in paper machine draws, the strain-rate is 10 s^−1^ and beyond (Kouko and Retulainen 2015).

Since paper is a viscoelastic material, it exhibits strain-rate dependent mechanical properties. Therefore, it is crucial to investigate the mechanical behaviour of paper at different strain-rate levels. In the literature, the mechanical behaviour at low strain-rates was investigated (Andersson and Sjöberg 1953; Östlund and Niskanen 2021; Szewczyk et al. 2016). At higher strain-rates there is only elastic stiffness available from ultrasonic testing (Baum and Bornhoeft 1979; Mann et al. 1980), however, since processing operations such as converting are taking place at high rate levels, it would be desireable to also capture deformation beyond the elastic regime and material failure. Furthermore, considering the viscoelastic nature of paper, the stiffness and strength values of the material are underestimated in the low strain-rate tests reported in the literature. Thus, the presented work is relevant for working out better material models and more realistic material parameters for paper converting operations.

#### Background strain-rate effects and viscoelasticity

When increasing the rate of loads causing strains in materials, their mechanical behaviour in terms of stiffness, yield strength and failure strain will change in comparison to a pre-defined reference load rate. In most engineering materials a strain-rate hardening is observed, causing higher strength with increasing loading rate. Three regimes are observed: (1) Regime I, the weak sensitivity area, (2) Regime II, the strong sensitivity area, and (3) Regime III, the saturation area. In the first regime, strength (and stiffness) slowly increases with strain-rate, in the second, strength and stiffness increases rapidly, until, in the third, the sensitivity to strain-rate decreases again. This was shown for rocks (Qi et al. 2009), metals (Zhang et al. 2012) and polymers (Al-Maliky and Parry 1994; Gerlach et al. 2008; Hu et al. 2006; Sato et al. 2008). The three regimes are bounded by two transition strain-rates or turning points. The first turning point was estimated to be 100 s^−1^ in brittle materials (rock and stone), roughly 300 s^−1^ in synthethic polymers and 1000 s^−1^ in metals (Yu et al. 2013). However, other authors name lower values, e.g. for brittle materials like concrete with a turning point at 5 s^−1^ (Houqun et al. 2016), or 100 s^−1^ in synthethic polymers (Jordan et al. 2008; Siviour et al. 2005; Siviour and Jordan 2016).

The experimental means to study the behaviour in these regimes are diverse, covering universal testing machines (Regime 1), Split Hopkinson bars (Regime 2), and light gas, rail or plasma guns (Regime 3), see (Meyers 1994). While, positive strain-rate hardening is common, in most materials, a negative strain-rate hardening was observed, e.g. in bacterial cellulose (BC) nanopaper (Santmarti et al. 2020).

Over the last decades multiple constitituive laws have been established to describe the behaviour of materials exhibiting strain-rate hardening. However, the mechanisms behind are still heavily debated (Kun et al. 2009) and they are manifold for different materials (Houqun et al. 2016; Kun et al. 2009; Siviour and Jordan 2016). These mechanisms do not occur exclusively, but they are acting in parallel, some of which become more prominent at certain strain-rates, temperatures or moisture contents. This explains also the non-linear behaviour of dynamic strength over strain-rate (Qi et al. 2009). One well established model for describing strain-rate hardening and strain-rate effects in general was proposed by Cowper and Symonds (Cowper and Symonds 1957), originally developed to scale the yield stress. For polymeric materials the model was also used to scale the elastic and strength property due to strain-rate (Pan et al. 2017). Here, a dynamic increase factor DIF [-] is defined by the strain-rate $\dot{\varepsilon }$ [s^−1^] and two coeffients D [s^−1^] and q [-], see Eq. 1 (Somarathna et al. 2020).

1 $$DIF=1+ {\left(\frac{\dot{\varepsilon }}{D}\right)}^\frac{1}{q}$$

A common approach for describing strain hardening behaviour, which is typically applied to metals, is the power law (Mukarati et al. 2019). The stress σ [MPa] is expressed by the strain ε [−] as well as by the strength coefficient K [MPa] and the strain hardening exponent n [−], see Eq. (2). The aforementioned parameters are intended to be represented as scalars. Erkkilä et al. used the power law approach, along with other plasticity models, to describe the stress–strain response of paper sheets (Erkkilä et al. 2013).

2 $$\sigma =K\cdot {\varepsilon }^{n}$$

In crystaline solids, e.g. metals widely accepted strain-rate mechanisms (Follansbee 1986; Klahn et al. 1970; Kun et al. 2009) are related to diffusion, dislocation movement, their interaction, barriers and drag (Follansbee 1986; Kun et al. 2009; Lindholm 1964; Ross 1997). In rock and soil, similar mechanisms are described, e.g. “macroviscous mechanisms” (Houqun et al. 2016; Qi and Qian 2003 cited from (Qi et al. 2009); Zhurkov 1965). Other phases or enclosed fluids can be the leading cause in strain-rate behaviour, such as air or water entrapped in cellular or porous materials (Li Piani et al. 2020). This is also the case in fibre networks, such as pulp, where the rate dependence is related to the squeeze out of intra-fibre water from the fibre-walls (Lobosco and Kaul 2001).

Polymer chains show a huge span in length scales, ranging from molecules (< 1 nm) over chain-segments (11 nm) to complete chains (> 11 nm). Each length scale has unique (vibrational) motion frequencies ranging from 10^–4^ to 10^10^ Hz. These vibrational motions affect the entire chain, causing polymers to show a time-dependent deformation, i.e. rate-dependency. The microscopic changes in deformation can be distinguished in the elastic bond stretching and chain straightening, but also in the non-reversible chain slippage and chain breakage (Cho 2016; Lin 2011; Shaw and MacKnight 2018). Individual pulp fibres exhibit a strain-rate dependency in stiffness, with an increase in longitudinal elastic modulus of 5% to 12% per tenfold increase in loading rate, see (Zizek et al. 2022). Consequently, in paper the viscoelasticity mainly stems from the fibre and not from bond-breakage (Seth and Page 1981), contrary to the original assumption of Rance (Rance 1948) cited from (Barkas et al. 1953). Notably, paper is not only viscoelastic. Its nonlinear stress–strain behaviour also arises from irreversible elongation, i.e. plasticity (Alava and Niskanen 2006) in combination with pronounced creep (Coffin 2009).

Similar to the dislocation theory in metals, where barriers to dislocations can increase the yield, there are motion restricting processes in polymeric chains that control the yield (Mulliken and Boyce 2006). The molecular motion can become restricted e.g. at certain temperatures or strain-rates, leading to an increased strength.

For cellulose, for example, it has been shown that intramolecular bond lengths, bond angles and dihedral angles are the main contributors to the strain of the system. As the rate decreases, the contribution of bond length changes decreases too (Jiang et al. 2023; Mulliken and Boyce 2006; Siviour and Jordan 2016).

Polymers exhibit a high strain-rate sensitivity zone, Regime II. This increase in strain-rate sensitivity can be attributed to the activation of a particular molecular mobility, e.g. side chain motion or ring flips (beta-transition) in glassy polymers, see (Siviour und Jordan 2016). In semi-crystalline polymers, which can be thought as a two-phase structure, containing rigid crystalline in a soft amorphous mass (Siviour and Jordan 2016), the behaviour is strongly dependent on the crystallinity—which depends on the temperature. On the other hand, the moisture sensitivity is affected by amorphous components, due to plasticization (decreasing stiffness with moisture content), see (Coffin et al. 2004).

The strain-rate behaviour of paper and wood products has been investigated—with a strong focus to creep and lower to moderate strain-rates—over the last eight decades (Barkas et al. 1953). Gibbon (Gibbon 1944), showed that slow loading leads to a distinct decrease in breaking load, but not in breaking stretch. Rance (Rance 1948) cited from (Barkas et al. 1953) showed that breaking load increased exponentially with decreasing load time, indicating a time-dependence of fracture load. Similarly, Jacobsen (Jacobsen 1950) cited from (Barkas et al. 1953) showed a linear relationship between the breaking load and the logarithm of the “time to failure”. The first to report on high strain-rate behaviour was published by Steenberg (Steenberg 1949). With impactor tests of newspaper (time to failure: 1 ms), he showed that an increase in strain-rate by 1 million led to an increase in break loads by 1000%. For rag-bond paper the increase was found to be only 60%. Similar to Rance (Rance 1948) cited from (Barkas et al. 1953) he reported only a marginal effect on breaking stretch due to rapid loading. Mason (Mason 1948) cited from (Barkas et al. 1953) showed (for hand-made sheets), that the shape of the stress–strain curve changes with strain-rate. At low rates, the curves show a concave-convex (upward inflexion after the yield point) shape, see (Barkas et al. 1953). With increasing rate, the convex upward inflexion vanishes (but downward inflexion becomes more pronounced). But not only the apparent tensile strength and tensile stiffness of dry paper increases with strain-rate, as reported in 1953 by Andersson and Sjöberg (Andersson and Sjöberg 1953), but also those of individual fibres (Hardacker 1970).

The effect of strain-rate on wet paper is a crucial property for the runnability. High breaking strength and strain provides a wider control window for web draws. The strain-rate was found to influence the shape of the stress–strain curve of the wet web. The higher the rate, the higher the failure strength, the lower the failure strain, the lower tension-to-tensile stiffness ratios and the higher the short-time relaxation rate (< 100 ms) of wet paper web, see (Green 1981; Kouko and Retulainen 2015). Wet paper was found to be particular sensitive to strain-rate (Alava and Niskanen 2006). Paper has a time-dependent mechanical response. Therefore, the stiffness obtained with ultrasonic methods is inherently high (by approx. 10%). The larger the moisture, the larger the strain-rate induced amplification (Coffin et al. 2004).

Zezin et al. (Zezin et al. 1984) investigated the strain-rate behaviour of cellulose base polymer materials with a thickness of 2.5 mm. Up to a rate of 200 s^−1^ the strength amplification was roughly 20%. The strain-rate sensitivity increased strongly—until it levelled off at approx. 500 s^−1^ to an amplification of 120%. With cellulose nanopaper a non-linear strain-rate effect was observed (Santmarti et al. 2020). At rates lower than 0.25 s^−1^, tensile strength and tensile modulus stayed constant, but decreased at higher rates. The decrease was stronger, the smaller the grammage of the paper. The decrease in strength and stiffness at higher rates was attributed to the inertial effect, i.e. that curled nanofibres fail to uncurl at higher rates. The observation that lower grammage paper sheets show a higher strain-rate softening, was attributed to the worse stress transfer between adjacent BC nanofibres.

#### High strain-rate testing with a Split Hopkinson bar

A Split Hopkinson bar is a common method for the high strain-rate mechanical testing of material samples in a strain-rate range of roughly 10^2^ s^−1^ to 10^4^ s^−1^ (Al-Mousawi et al. 1997; Chen and Song 2011; Shin and Kim 2019). It consists of several long and slender calibrated bars, called striker bar, incident bar and transmission bar, through which one-dimensional pulses are travelling. A pulse is generated in the striker bar section by different kinds of propulsion systems such as gas guns, pendulums, explosives, bar pre-loadings, etc. (Baumann et al. 2021). From the striker bar the pulse further travels through the incident bar until it reaches a material sample which is positioned between the incident bar and the transmission bar. When reaching the sample, part of the stress pulse is reflected and another part transmits through the sample into the transmission bar. In order to measure these stress pulses, strain gauges are attached to the bars (Gama et al. 2004). By evaluating these measuring signals the strain-rate related mechanical response of the sample material can be determined (Mohr et al. 2010).

Applying dynamic testing to a thin sheet-like material such as paper is not as straightforward as quasi-static tensile testing even if the resulting deformation is in the same range. Here, it is important to consider the so-called mechanical impedance (Rotariu et al. 2022), which is a product of sample cross sectional area, material density and speed of sound in the material. The key point is that the signal–noise ratio of the measurements deteriorates with increasing difference in impedance between the bars of the Split Hopkinson setup and the tested material. Paper is considered—mainly due to its sheet-like geometry and relatively low density—a low impedance material in terms of dynamic testing. Therefore, one can expect a large impedance mismatch between the usually metallic bars of a Split Hopkinson setup and the thin paper sample which results in a poor signal-to-noise ratio. To improve this, setups need to be adjusted in their design involving a more sensitive bar setup. This can be achieved by the use of polymer bars instead of metallic ones (Casem et al. 2003; Sawas et al. 1998, 1995; Zhao et al. 1997), which offer a much lower mechanical impedance. Another approach is to use relatively small bar cross-sections (Paul and Kimberley 2015; Zhu et al. 2021) or hollow bars (Chen et al. 1999; Feng et al. 2018), especially for the transmission bar section. When testing low impedance materials like paper, the transmission bar has to be very sensitive, because only a small portion of the incoming pulse will transmit through the sample and reach this bar.

#### Contactless measurements with digital image correlation (DIC)

DIC is a well-established optical, contactless method for evaluating the surface deformation of an object during a mechanical test (Jones and Iadicola 2018). In order to evaluate the sample it is necessary to ascertain that it has either a natural surface pattern or an applied one (Bomarito et al. 2017). Hereby it is of paramount importance that this pattern is capable of following the deformation of the actual sample (Jones and Iadicola 2018). Images of the test sample can be taken by a single camera (2D-DIC) or multiple cameras (stereo-DIC), see (Arza-García et al. 2022; Murienne and Nguyen 2016; Pan 2018). Digital image sequences captured during the test can be correlated using software tools to calculate deformation and strain fields, which are evolving over time. Regarding the type of software local and global DIC methods can be distinguished (Blaysat et al. 2020; Wang and Pan 2016). In the local approach the solution at a given point of interest is contingent upon a small subset of the image in its vicinity. In contrast, in the global approach, the solution at a specific point is also contingent upon the solutions of other points in its vicinity. The use of DIC is independent from the loading velocity and can be employed universely. There are numerous examples in the existing literature werein this method has been applied to Split Hopkinson bar tests (Chen et al. 2014; Hammer et al. 2015; Meyland et al. 2023; Pandya et al. 2019).

### Aim of this work

The objective is to apply a suitable test method for determining the strain-rate dependent material characteristics of paper using a Split Hopkinson test bench. In combination with quasi-static testing, the strain-rate dependent tensile behaviour of paper sheets of 160 g/m^2^ grammage is investigated over a range of four to five orders of magnitude ranging from 0.0083 to 212 s^−1^ for the first time. All strain-rate related strength and stiffness properties of the paper are characterized as well as the influence of the strain-rate levels on the breaking strain. We are employing the Cowper-Symonds model to quantify the strain-rate hardening of the material. Furthermore, by recording the experiments with a high-speed camera and utilizing digital image correlation (DIC), the failure behaviour of the paper samples is investigated and relevant differences to failure under quasi-static tension are discussed.

## Materials & methods

### Sample preparation

An industrially produced softwood Kraft pulp (Mondi Frantschach, Austria), which was unbleached and unrefined was used as the paper material. It was made from a mixture of spruce and pine, having a kappa number of 42. The pulp was treated in a valley beater for 60 min reaching a beating degree of 24.7 ± 0.6°SR (i.e. 509 ± 10 CSF) according to ISO 5267/1, and a water retention value (WRV) of 1.3 g/g according to ISO 23714. A Rapid Köthen handsheet former was used to produce the sheets in accordance with ISO 5269–2. These handsheets had a caliper of 223 ± 7 (ISO 534) µm and a grammage of 160.1 ± 1.8 g/m^2 ^(ISO 536).

In Table 1, the tensile parameters determined for the papers from 30 individual strips with standard testing procedures are summarized (ISO 1924–3:2005).

**Table 1:** Mechanical parameters from the standard testing of the papers: Tensile force, Tensile index, Tensile stiffness index (TSI), Tensile energy absorption (TEA), TEA index, breaking length, and breaking strain. All the values are given as mean ± standard deviation of 30 individual samples

Tensile force [N]	Tensile Index [Nm/g]	TSI [kNm/g]	TEA [J/m^2^]	TEA index[mJ/g]	Breaking length [km]	Breaking strain [%]
161.5 ± 17.9	67.2 ± 7.4	8.5 ± 0.3	162 ± 48	1010 ± 300	6.86 ± 0.8	2.12 ± 0.41

**Fig. 1:**
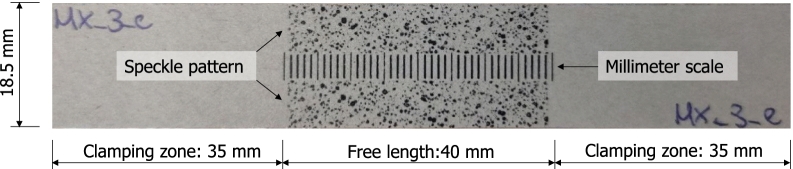
Paper sample prior to mechanical testing, including its dimensions

Handsheets were cut into 110 mm long test samples with a width of (18.5 ± 0.2) mm for the quasi-static and dynamic tests. The free span length between the clampings was 40 mm. Deformation as well as the strain-distribution in the sample during dynamic testing was evaluated from digital image correlation analysis of high speed camera images, hence a millimeter scale and a speckle pattern was printed on the samples. Printing was done using xerography in order to not alter the mechanical paper properties due to wetting. In Fig. 1, an exemplary paper sample is presented.

### Quasi-static testing

Quasi-static tensile testing of the paper samples was carried out using a Universal testing machine (Zwick Roell, Z010, Germany). Here, the testing was performed in a climate chamber (480X, RU MED—Rubarth Apparate GmbH, Germany) at a temperature of 23 °C and a relative humidity of 70% RH. This relative humidity was chosen to match the RH during the Split-Hopkinson tests, where it was not possible to control RH. Prior to the testing, the paper samples were conditioned in this environment for at least 12 h. Displacement-controlled tensile tests were performed at three different strain-rates—0.0083 s^−1^ (equivalent to 20 mm/min, the standard rate), 0.042 s^−1^, and 0.42 s^−1^, see Table 2. Fifteen samples were tested for each strain-rate. It should be mentioned that the sampling frequency of those samples tested at 0.42 s^−1^ was insufficient when it comes to the determination of the exact maximum force and failure strain. As the sampling frequency of the instrument was not adaptable, the number of data points for the highest testing speed at 0.42 s^−1^ was limited.

**Table 2:** Overview of the loading velocities and the associated strain-rates of the quasi-static tests

Loading velocities [mm/min]	20	100	1000
Strain-rates [s^−1^]	0.0083	0.042	0.42

### Dynamic testing

Based on the results from the numerical study, which can be found in (Baumann et al. 2024), the decision was made to use an aluminium based Split Hopkinson setup including a hollow and tapered transmission bar. The residual wall thickness in the tapered area of the transmission bar is 1.0 mm, resulting in a cross-section of 47.1 mm^2^. This is roughly seven times smaller than the cross-section of the solid striker and the incident bar with 314.2 mm^2^. Therefore, the hollow transmission bar is much more sensitive than a comparable solid bar. Furthermore, the sample holder design is asymmetric with a heavier one on the solid incident side and a lighter one on the hollow transmission side. These measures together with aluminum as a friction liner material would result in the most promising arrangement, according to the numerical study.

**Fig. 2:**
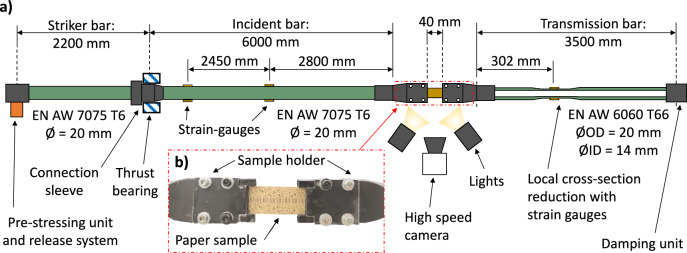
Schematic sketch of the Split Hopkinson test bench (**a**) and detailed photo of the paper sample and the clampings positioned between the incident and the transmission bar (**b**)

A schematic sketch of the experimental test setup as well as a detailed photo of the paper sample and the clampings can be seen in Fig. 2. The test bench is propelled by an elastically pre-stressed striker bar, where the energy is quickly released by a specially designed mechanism, see (Baumann et al. 2021). In the present tensile test setup the striker bar and the incident bar are connect by a sleeve to allow the transmission of a tension stress pulse. The paper sample is fixed between the sample holders, which in turn are threaded to the incident and transmission bar end. The samples were quasi-statically pre-tensioned with a force of 12.5 N prior to the actual test, to guarantee reproducible starting conditions. The testing environment was under ambient conditions (T ~ 24 °C, RH ~ 67%). With the Split Hopkinson bar five samples were tested for each strain-rate. Only for the highest strain-rate eight samples were examined.

The incident bar and the transmission bar are equipped with strain gauges. A “Dewe3-M4” amplifier combined with a “Trion-1820-Multi-4-D-Card” (Dewetron, Grambach, Austria) was used to sample the strain-gauges at a rate of 2 MHz. Approximately 0.9 ms is the maximum possible test duration with the shown bar setup. The actual test duration depends on the selected bar velocity and the failure strain of the sample and can be as low as 0.15 ms at the highest bar velocity. Data processing and analysis were performed with the software Diadem 2020 (National Instruments, Austin, TX, USA). A CFC 15,000 low pass Butterworth filter was used to eliminate the background noise. As the strain-gauge equipped bars are calibrated, the determined strains can be converted into forces and stresses.

Apart from the actual measuring signals high-speed images of the material samples were taken at 50 kfps with a shutter time of 4 μs using a “Phantom Veo 640” (Ametek, Berwyn, IL, USA). The deformation of the paper was evaluated using two different analysation methods. In the first approach, the one-dimensional extension or strain in loading-direction was evaluated by target tracking of the imprinted ruler markers (millimeter lines). This was done by using the open-source software package Blender version 2.93. (Blender Foundation, Amsterdam, Netherlands). The second approach was to analyse the strain-distribution two-dimensionally by tracking the imprinted speckle pattern. The size of the speckle pattern ranged from roughly 0.1 to 0.8 mm. For post processing of the images, the software package GOM Correlate 2022 (Zeiss, Oberkochen, Germany) was used.

The dynamic experiments were carried out using five different loading velocities. These velocities ranged from roughly 2.7 m/s up to 13.6 m/s. An overview of the loading velocities and the associated strain-rates is given in Table 3.

**Table 3:** Overview of the loading velocities and the associated strain-rates of the dynamic tests

Loading velocities [m/s]	2.7	3.6	6.3	10.0	13.6
Strain-rates [s^−1^]	61.7	81.8	112.3	166.6	211.9

Together with the quasi-static experiments this results in a total of eight different loading velocities or strain-rates ranging from 0.0003 to 13.6 m/s.

## Results and discussion

**Fig. 3:**
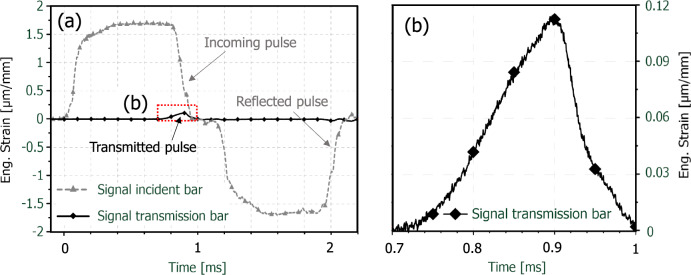
Overview of exemplaric unfiltered raw signals from the first incoming, reflected and transmitted pulse at a loading velocity of 13.6 m/s (**a**) and detailed view of the transmitted signal (**b**)

In order to give an impression of the relation between the incoming, reflected and transmitted pulse exemplaric unfiltered measuring signals are shown in Fig. 3. It can be clearly seen that due to the low impedance of the paper sample the transmitted signal is much weaker as the incoming and reflected one, see Fig. 3(a). Nevertheless the signal clearly stand out from the background noise, as can be seen in Fig. 3(b). If the transmission bar would have been solid like the striker and the incident, the resulting raw signal would have been considerably weaker.

### Stress–strain characteristics

**Fig. 4:**
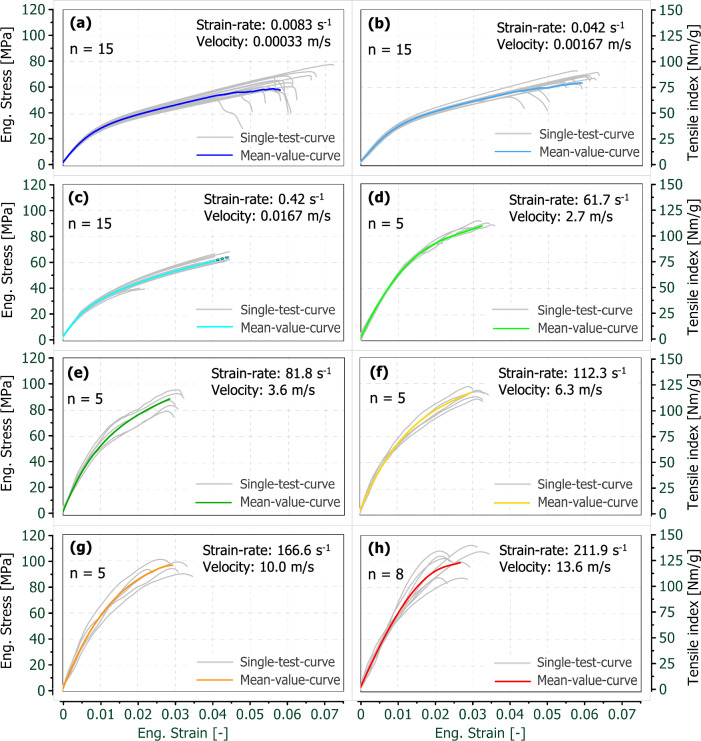
Overview of the stress–strain or tensile index-strain curves subdivided by their strain-rate levels **(a**) to **(h)**, including the single test curves (grey) and the mean value curves (coloured)

In Fig. 4 the stress–strain or tensile index-strain curves are shown for each of the eight strain-rate levels. These strain-rate levels range from 0.0083  up to about 212 s^−1^. Apart from the single test curves, also averaged curves were evaluated, calculated as described in (Klug et al. 2019). Comparing the curves, it is clearly visible that both the maximum stress as well as the initial stiffness are increasing with increasing strain-rate. The failure strain, however, is behaving the opposite way and is decreasing with increasing strain-rate. Regarding the variation of the failure strain, it is on average smaller for the dynamic strain-rates.

As mentioned in the Materials & Methods section, the sampling frequency of the quasi-static testing device was limited. To account for this inaccuracy, the mean value curve of the 0.42 s^−1^ strain-rate experiments was extended with a dotted line. Hereby, the length was estimated by the average gap between the last two data points which is roughly 9% of the total strain. Nevertheless, it can be clearly seen in Fig. 4(c) that the fracture strain at 0.42 s^−1^ is conceivably lower than at 0.0083 s^−1^ and 0.042 s^−1^, see Fig. 4(a), (b).

**Fig. 5:**
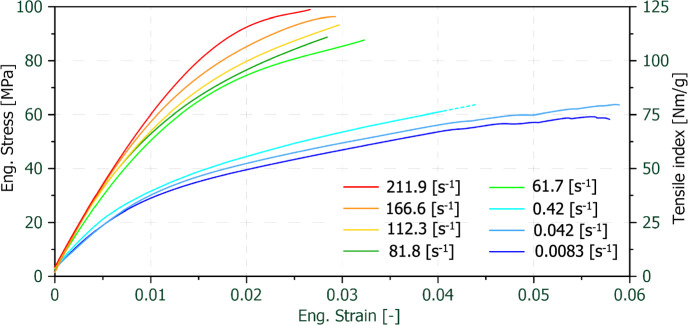
Averaged curves of the engineering stress–strain or tensile index–strain data for all strain-rates

A comparison of the mean value stress–strain or tensile index-strain curves is shown in Fig. 5. As can be seen, the individual curves form two clusters in this plot. The three lower strain-rates exhibit similar tensile strength and initial stiffnesses. The same is visible for the five mean value curves at higher strain-rates. In addition, the general curve characteristic can be described as almost bi-linear for the lower strain-rates, which means that they have a primary and a secondary stiffness or section. With increasing strain-rate, the stress–strain curve becomes more linear, meaning that the primary section increases while the share of the secondary section gets smaller. While the mean fracture strain at the two lowest strain-rates is close to 6% it decreases to roughly 4% to 4.5% for 0.42 s^−1^. It drops even further to around 3% at high strain-rates.

**Fig. 6:**
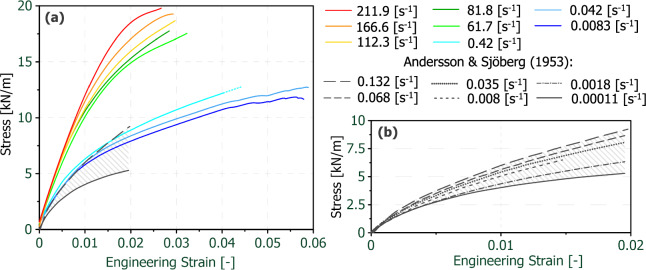
Comparison of the stress–strain curves with strain-rate related data from Anderssson and Sjöberg (Andersson and Sjöberg 1953) cited from (Östlund and Niskanen 2021); comparative display including only their min. and max. strain-rate curves (**a**) and detailed view of all curves from Andersson and Sjöberg (**b**)

In the literature, stress–strain curves of tensile tests on paper sheets covering a range of 0.00011 s^−1^ to 0.132 s^−1^ were reported (Andersson and Sjöberg 1953; Östlund and Niskanen 2021). A comparison of the stress- strain curves from literature with the own results can be seen in Fig. 6(**a**). A detailed view of the curves reported in (Andersson and Sjöberg 1953; Östlund and Niskanen 2021) is given in Fig. 6(**b**). It can be noticed that the four higher strain-rates (0.008 s^−1^ to 0.132 s^−1^) from Andersson and Sjöberg (Andersson and Sjöberg 1953) are in a similar range as the ones from the own quasi-static tests (0.0083 s^−1^ to 0.42 s^−1^). A good agreement can be observed by a strain-rate wise comparison of the stress–strain characteristics of these two datasets.

**Fig. 7:**
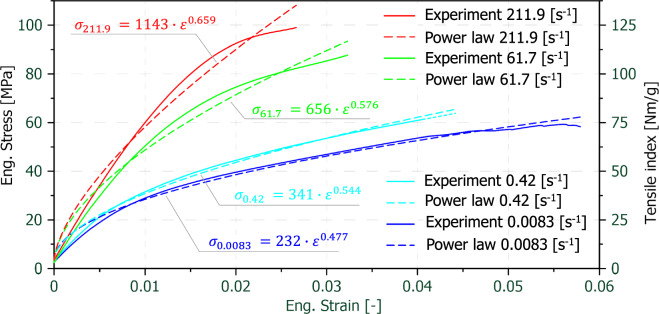
Comparison of the experimentally obtained mean-value curves (solid lines) with the power law fits (dashed lines) for the lowest and highest quasi-static and dynamic strain-rates

The power law approach was used to describe the stress–strain-relationship of the mean value curves for each of the tested strain-rates, compare Eq. (2). In order to find the best possible fit, the strength coefficient K and the strain hardening exponent n were solved using the least square method. In Fig. 7 a comparison of the experimentally obtained mean value curves (solid lines) with the power law fits (dashed lines) can be seen. For clarity reasons only the lowest and highest quasi-static strain-rate curves as well as the lowest and highest dynamic strain-rate curves are plotted. The power law model appears to provide a good overall fit for all strain-rates, although it overestimates the initial stiffness.

#### Strain-rate dependent strength, stiffness, energy absorption and model fits

**Fig. 8:**
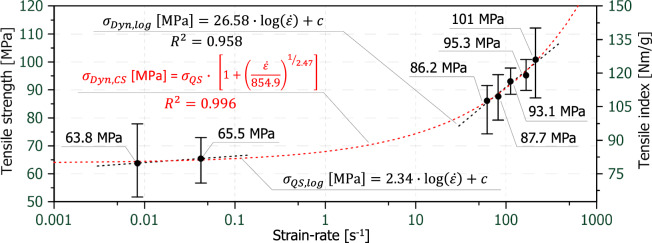
Mean tensile strength and mean tensile indexes including the scatter bands over strain-rate (logarithmic display), error bars are the 95% confidence limits

Figure 8 shows the mean tensile strength or tensile index values for all strain-rates including the scattering on a semi-logarithmic scale against the strain-rate. Due to its limited accuracy from the slow sampling rate, the data point at 0.42 s^−1^ was not included. It can be seen that, initially, the increase in tensile strength is rather flat at low strain-rates but gets steeper by roughly eleven times at the five high strain-rates. The mean tensile strength value at the highest strain-rate (101 MPa) is around 58% higher than the mean tensile strength at the lowest strain-rate (about 64 MPa). In order to describe the strain-rate related tensile strength increase, the Cowper-Symonds model was used, compare Eq. (1). The optimum coefficients (D = 854.9 and q = 2.47) for the model were determined using the least square method. This resulted in a relatively good fit with a coefficient of determination (R^2^) of 0.996.

**Fig. 9:**
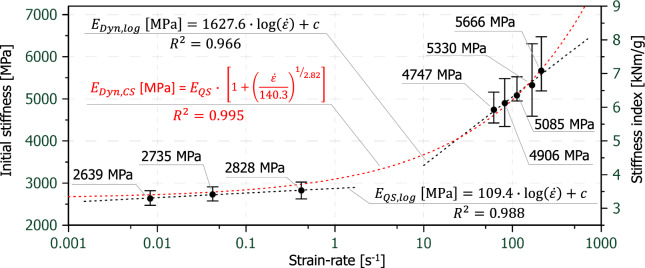
Mean values of the initial stiffnesses and mean stiffness indexes including the scatter bands over strain-rate (logarithmic display), error bars are the 95% confidence limits

Next, the mean values of the initial stiffness and mean stiffness index values are presented on a semi-logarithmic scale against the strain-rate in Fig. 9. In practice, the stiffness of paper samples is evaluated by using the maximum gradient in the linear range of the force-strain curve. Due to the fact that the curves at elevated strain-rates are a bit uneven and wavy and not as continuous as in quasi-static tests, it was decided to evaluate all stiffnesses as the gradient between 0 and 1% strain. In contrast to the previous results for the tensile strength and the tensile index, the 0.42 s^−1^ strain-rate was included since this part between 0 and 1% strain of the stress–strain curve is accurately captured. Again, the trend is similar to Fig. 8, the increase in stiffness is much steeper at high strain-rates than at low strain-rates. Similar to the strain-rate related strength properties also the stiffness increase can be described with a Cowper Symonds model. An optimization of the coefficients (D = 140.3 and q = 2.82) with the least square method resulted in a coefficient of determination (R^2^) of 0.995.

In summary, Figs. 8 and 9 show that a dynamic amplification based on an exponential function can be used to fit strength and stiffness over a wide range of strain-rates reasonably well with an R^2^ exceeding 0.995. Fits with a logarithmic law (not displayed) have returned poorer R^2^ values.

**Fig. 10:**
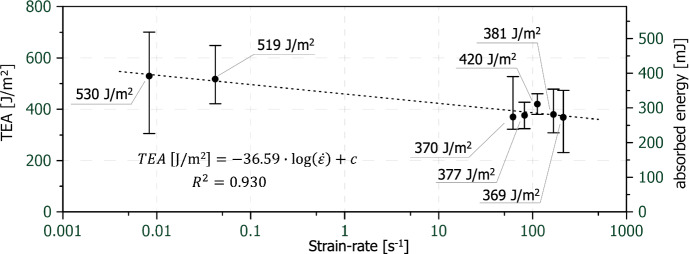
Mean values of the TEA and absorbed energy including the scatter bands over strain-rate (logarithmic display), error bars are the 95% confidence limits

In Fig. 10, the tensile energy absorption (TEA) and absorbed energy are plotted, again on a semi-logarithmic scale against the strain-rate. The TEAs and absorbed energies were integrated to the point of final material failure. As in Fig. 8 the data point at 0.42 s^−1^ was not included due to its limited accuracy. The overall trend is linearly decreasing from a mean TEA value of 530 J/m^2^ at lowest strain-rate to roughly 370 J/m^2^ at the highest strain-rate, which corresponds to a decrease of about 30%. This decrease demonstrates that the drop in strain at break affects TEA and absorbed energy more than the increase in tensile strength (compare Fig. 5). Szewczyk et al. (Szewczyk et al. 2016) reports an opposing trend for the TEA in almost creep-like to quasi-static testing at strain-rates between 0.42** × **10^–5^ to 0.042 s^−1^. However, it should be noted that the strain-rate range in the present study starts at quasi-static levels and goes up to high dynamic testing conditions (0.0083 s^−1^ to 212 s^−1^). This range differs significantly from the study mentioned before. Literature exists on the strain-rate related energy absorption of polymers loaded under tension at similarly high strain-rates. Govaert and Peijs (Govaert und Peijs 1995) performed tests on polyethylene fibres in a strain-rate range of 10^–5^ s^−1^ to 10^2^ s^−1^. According to their study the work of fracture is decreasing with increasing strain-rate. Wang et al. (Wang et al. 2021) did tests on polyurethane acrylate (PUA) samples at strain-rates ranging from 0.001 to 1000 s^−1^. Their tensile test results showed that the fracture strain at quasi-static strain-rates is one order of magnitude higher than at dynamic strain-rates. Therefore, also the absorbed energy drops as the strain-rate increases. Other tensile tests on glass fibre and carbon fibre reinforced polyamide-6 at strain-rates from 10^–2^ s^−1^ to 40 s^1^ were conducted by Todo et al. (Todo et al. 2000). While the failure strain and the absorbed energy of some composite types increased with increasing strain-rate also opposite trends were observed at a certain point.

**Fig. 11:**
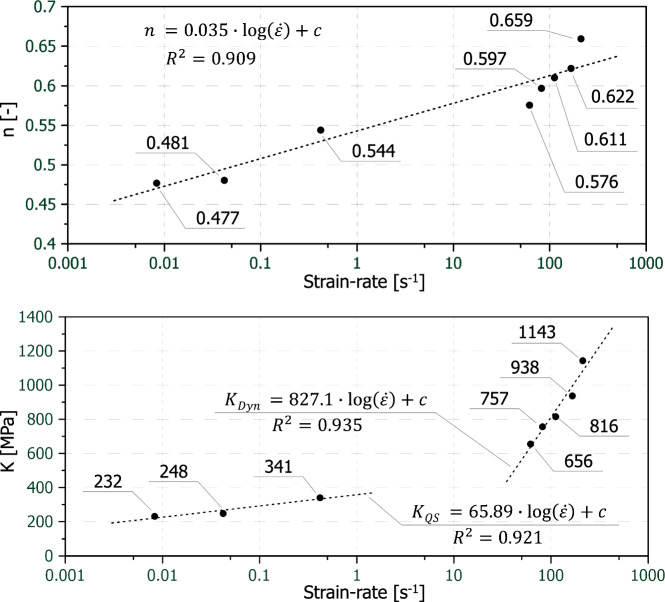
Comparison of the strength coefficients K and the strain hardening exponents n from the power law approach over strain-rate (logarithmic display)

As can be seen in Fig. 11 the strength coefficient K as well as the strain hardening exponents n from the power law approach are both increasing with increasing strain-rate. The increase of exponent n means that the stress–strain-curves are becoming more linear as the strain-rate increases. The fact that n is not constant means that a power law (Eq. 2) describing strain hardening cannot simply be scaled multiplicatively by a dynamic increase function (Eq. 1) to account for strain-rate hardening.

It must be noted, that the strength coefficient K must not be considered equal to the modulus E (Fig. 9) nor the tensile strength (Fig. 10). The first is only the case, when n equals 1. It is further stressed, that no unified strain- and strain-rate hardening model was established, i.e. Eq. 1 and Eq. 2 were not unified, but analysed independently.

#### Strain-distributions and fracture patterns

**Fig. 12:**
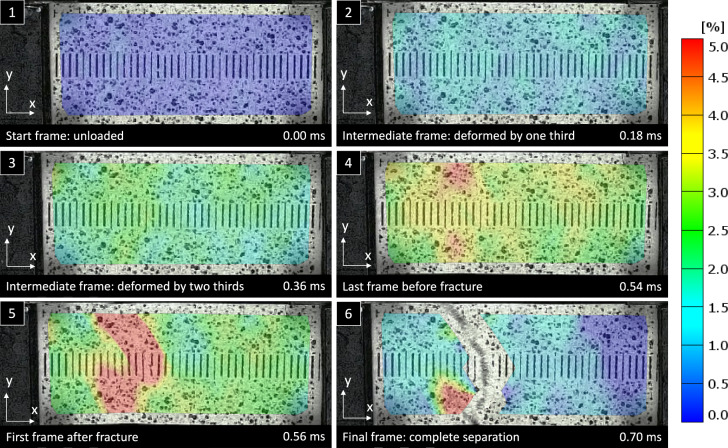
Exemplary high-speed sequences from the DIC analysis of a paper sample tested at 2.7 m/s or 62 s^−1^ on the Split Hopkinson test bench showing the strain distribution (ε_xx_) in loading direction

In Fig. 12, different high-speed sequences of an exemplary sample with the according time steps are illustrated. The strain distribution of the sample in loading direction (ε_xx_) is presented at a strain-rate of about 62 s^−1^ using DIC. In the first loading stages, there is a rather uniform strain-distribution. Shortly before first cracks are initiated, there are some strain-concentrations noticeable towards the edges. The full experiment lasts less than 1 ms, and the fracture starts at 0.56 ms after loading. The pixel noise observed in the high-speed videos was found to be insignificant, with a range of ± 0.2% and no discernible impact on the physical strains. Overall, the main failure mechanism was fibre pulling which is caused by the failure of the fibre–fibre bonds within the paper, which is also clearly visible in the optical images in Fig. 14.

**Fig. 13:**
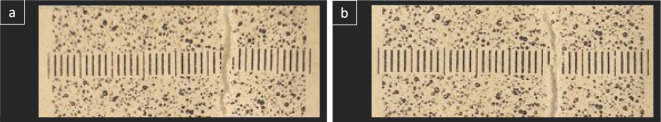
Representative failure images of two paper samples (**a**) and (**b**) after quasi-static testing at 0.0083 s^−1^

**Fig. 14:**
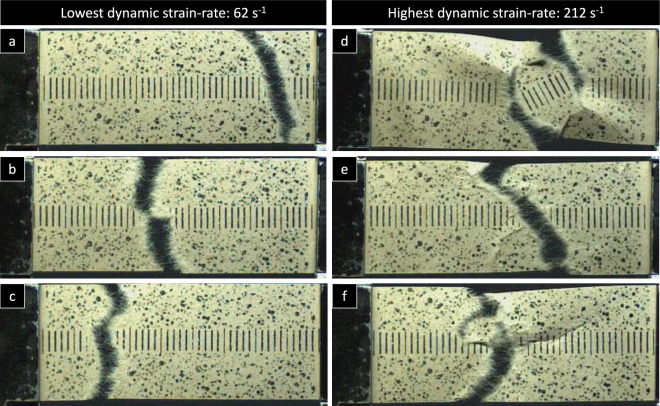
Representative failure images of the paper samples at the lowest dynamic strain-rate (**a**)–(**c**) and the highest dynamic strain-rate (**d**)–(**f**) captured during the test with the high-speed camera

In Fig. 13, the failure of paper samples after quasi-static testing at 0.0083 s^−1^ show the expected rather straight fracture. A similar fracture behaviour is also found for the lowest strain-rate of 62 s^−1^ of the dynamic testing, see Fig. 14(a)–(c). However, for the highest strain-rate of 212 s^−1^, the failing of the paper samples is much more discontinuous and rather resembles rupturing. In Fig. 14(d)–(f), a few representative cases at 212 s^−1^ are presented.

These findings are suggesting that at high strain-rates the failure mechanism of paper under tensile load is changing quite fundamentally. Under quasi-static conditions strain localization is a highly important characteristic (Considine et al. 2024). There regions with lower grammage or fibre orientation are starting to develop higher strain nearly immediately after start of the tensile experiment, long before the onset of fibre failure (Lahti et al. 2020). Considering how late the strain starts to localize at the fracture point, Fig. 12, and how frequent multiple failure spots in one sample can be found in Fig. 14, it can be concluded that strain localization is loosing its relevance as a failure mechanism at high strain-rates. As a consequence of that it can further suggest that the strain localization observed for quasi-static testing can be attributed to viscous material behaviour, i.e. short term plastic creep, which does not have enough time to develop at high strain-rates.

## Conclusions

It has been demonstrated that it is possible to perform high strain-rate tensile tests on paper samples using a Split Hopkinson test bench with a hollow and tapered transmission bar. The paper samples tested, showed a mean increase in tensile strength of approximately 58% between the lowest strain-rate (0.0083 s^−1^) and the highest strain-rate (212 s^−1^). Hereby, the increase per decade turned out to be much steeper in the higher strain-rate range than it was at quasi-static levels. When it comes to the mean increase of the initial stiffness (evaluated between 0 and 1% strain) it was even more pronounced than the tensile strength. The mean initial stiffness at the highest strain-rate (212 s^−1^) was almost 115% higher than at the lowest strain-rate (0.0083 s^−1^). Like the tensile strength the relation between stiffness and strain-rate is characterized by a gentle increase at lower strain-rates and a steep increase at elevated strain-rates. The Cowper-Symonds model for strain-rate hardening provides a good description of both, the increase in strength and stiffness with respect to strain-rate. When it comes to the strain-rate related fracture strain it behaves quite opposite to the tensile strength and initial stiffness. While the fracture strain at quasi-static strain-rates is around 6% it decreases to roughly 3% in the dynamic strain-rate range. Furthermore, the stress–strain characteristics which can be approximated as bilinear at low strain-rates (primary and secondary section) become increasingly linear, meaning that the secondary section is almost not existent at the highest strain-rate. This was also shown by comparing the power law coefficients with respect to strain-rate. The comparison of the fracture patterns, from post-test and high-speed images, revealed some changes in the fracture angle between low and high testing velocities. While the fracture angle at quasi-static strain-rates is almost orthogonal to the loading direction, there is a pronounced slope, sometimes coupled with several crack initiation points, at high dynamic strain-rates. Thus we suggest that strain localization, which is highly relevant at tensile failure in quasi-static tests, is related to short term plastic creep of the material as it nearly entirely disappears at high loading rates of paper.

## Data Availability

The data that support the findings of this study are available from the corresponding authors upon reasonable request.
